# In this issue: Volume 114, Issue 4, April 2023

**DOI:** 10.1111/cas.15410

**Published:** 2023-04-02

## EGFR inhibition in EGFR‐mutant lung cancer cells perturbs innate immune signaling pathways in the tumor microenvironment

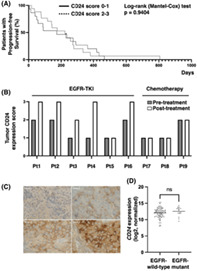

Lung cancer contributes towards a large proportion of cancer‐related deaths across the globe, making it a cause of growing concern. In non‐small cell lung cancer (NSCLC), mutations are frequently observed in genes encoding epidermal growth factor receptor (EGFR). Inhibitors that specifically target the mutated receptors, known as EGFR‐tyrosine kinase inhibitors (TKIs), thus exert strong control over cell cycle arrest in EGFR‐mutant NSCLC cells, and are being increasingly utilized in the treatment of NSCLC. However, successful EGFR‐TKI treatment remains challenging, as research shows that many patients develop resistance to treatment over time, ultimately leading to tumor relapse. The mechanisms through which EGFR‐TKIs may contribute to the immune escape of EGFR‐mutant cells have remained largely unexplored.

In this study, Shiiya et al. reveal that immune escape after EGFR inhibition in patients with EGFR‐mutant NSCLC may be a consequence of alteration of the tumor microenvironment. Additionally, the accelerated release of cell‐free DNA (cfDNA) upon EGFR inhibition triggers an innate immune response in the neighboring immune cells, leading to increased resistance to EGFR inhibition in tumor cells.

Using in vitro assays and patient samples, the researchers found that EGFR‐mutant cells had enhanced CD24 expression following EGFR‐TKI treatment. Interestingly, in the presence of anti‐CD24 antibodies there was an increase in the number of macrophages targeting tumor cells treated with EGFR‐TKI. This bolsters the potential of CD24 as a therapeutic target in the treatment of EGFR‐mutant lung cancer.

Taken together, these findings highlight that targeting the innate immune system using anti‐CD24 antibodies and monitoring cfDNA can help counter EGFR inhibitor resistance in NSCLC cells and improve clinical outcomes.

https://onlinelibrary.wiley.com/doi/10.1111/cas.15701

## Silencing of OGDHL promotes liver cancer metastasis by enhancing hypoxia inducible factor 1 α protein stability

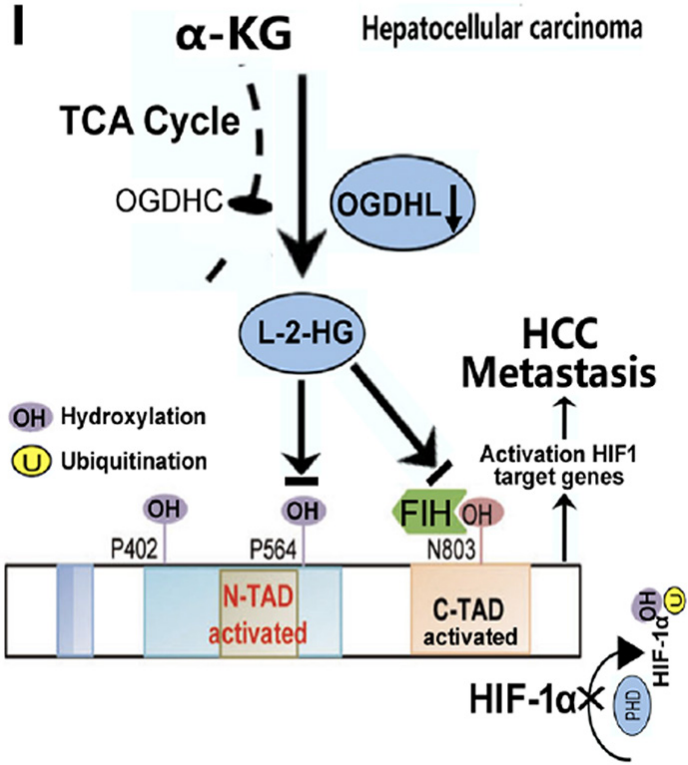

Hepatocellular carcinoma (HCC) is a common malignant disease and one of the leading causes of cancer‐related deaths globally. It progresses very rapidly, thus getting diagnosed only in the advanced stages.

HCC metastasis is driven by epithelial‐mesenchymal transition (EMT), a process through which epithelial cells transform into cancerous cells. One of the key triggers of EMT is hypoxia‐inducible factor‐1 (HIF‐1), which in turn is regulated by HIF‐1α protein expression. Recent studies have confirmed that HIF‐1α concentrations are directly related to HCC metastasis, and have revealed the involvement of smaller regulatory molecules, which have not been identified yet.

Additionally, previous studies have also identified the role of OGDH (the E1 component of oxoglutarate dehydrogenase‐like enzyme complex) in stabilizing HIF‐1α. However, the role of its isoform, OGDHL—which is downregulated in primary HCC tissues, has not been examined.

In this study, Dai et al. investigated the dynamics between HIF‐1α and OGDHL in HCC cells and its impact on cancer metastases. They analyzed samples from 281 HCC patients grouped in three independent cohorts.

They found low OGDHL expression levels in metastasized tumors. Further analyses of OGDHL levels in different HCC cell lines confirmed that low OGDHL concentrations were associated with invasiveness and metastasis of HCC, whereas high levels of OGDHL effectively inhibited these processes.

Next, they analyzed the correlation between OGDHL and HIF‐1α. Interestingly, OGDHL and HIF‐1α were found to be inversely related, suggesting that low OGDHL concentrations results in the accumulation of HIF‐1α, which in turn promotes EMT and HCC metastases.

The authors also investigated the mechanism by which OGDHL regulates HIF‐1α. To investigate this, they performed luciferase assays, which help determine whether a protein can activate or suppress a target gene. The researchers observed that low OGDHL levels increased the stability and transcription rate of HIF‐1α.

Moreover, fluorometric assays revealed that this increased stability of HIF‐1α was linked to high levels of (L)‐enantiomer of 2‐hydroxyglutarate (L‐2‐HG), a metabolite that prevents structural change in HIF‐1α. Furthermore, abnormally high OGDHL levels were found to successfully inhibit invasive growth and HCC metastasis in nude mice (mice with low immunity), suggesting that OGDHL maybe a major factor regulating HCC metastasis.

These results indicate that OGDHL‐regulated EMT can be a potential target for novel effective strategies for prevention and treatment of HCC.

https://onlinelibrary.wiley.com/doi/10.1111/cas.15540

## Suppression of androgen receptor signaling induces prostate cancer migration via activation of the CCL20–CCR6 axis

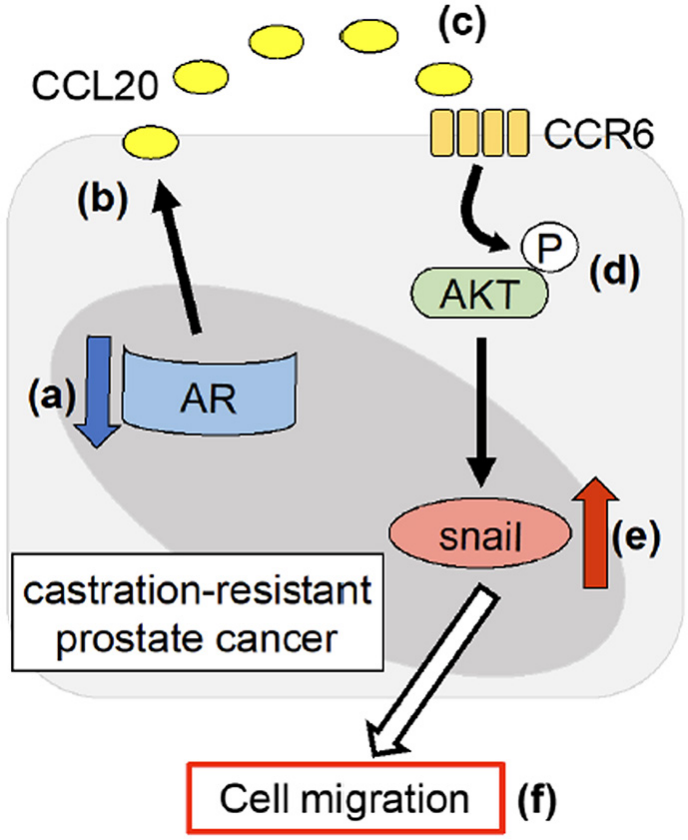

Androgen deprivation therapy is a standard approach for treating patients with advanced prostate cancer. However, in the long run, suppression of the androgen receptor (AR) signaling induces metastasis by increasing the migration potential of prostate cancer cells. Previously, research has demonstrated that chemokines and their receptors play an important role in activating the migration and growth of cancer cells in the tumor microenvironment. Moreover, the specific correlation of the C‐C chemokine ligand 20 (CCL20) and its receptor CCL6 with prostate cancer growth and adhesion has also been observed in the past. However, it is not clear if this chemokine‐receptor mechanism is involved in tumor migration on AR suppression.

In this study, Kano et al. sought to clarify this association by investigating the mechanism involved in the suppression of AR signaling in castration‐resistant prostate cancer. To this end, they conducted an in‐vitro transwell cell migration assay, using prostate cancer cells that pass through the transwell membrane and migrate to the lower chamber—also known as migratory cells.

They found that the expression levels of CCL20 were notably higher in the migratory (mig) cells, than in the parental (or prt) cells. In contrast, the levels of CCR6 (the receptor of CCL20) remained unchanged.

On further analysis, they found that the suppression of AR signaling promoted the secretion of CCL20, which ultimately increased the migration potential of cancer. To further explain the correlation between enhanced migration and suppression of AR, the authors blocked AR expression using three small interfering RNA (siAR) in LNCaP and C4‐2B cells. Increased cell migration was observed in the mig cells, siAR cells, and CCL20‐treated cells, along with low AR levels and increased CCL20 expression levels.

Since the AKT pathway is commonly upregulated in all three types of cells, the team also studied the AKT pathway downstream of the CCL20‐CCR6 axis. The AKT pathway is the passage point for various cytokine signals, and CCL20 is associated with the AKT pathway in other cancers. AKT inhibitors were found to suppress metastasis, indicating that the CCL20‐CCR6 axis induced cell migration in prostate cancer.

Analysis of immunostaining of human prostate cancer biopsy specimens revealed an association between metastatic potential, CCL20, and Snail expression (downstream of the AKT pathway).

This association was further confirmed using xenograft mouse tumor models, which revealed suppression of Snail expression with anti‐CCL20 antibody.

To sum up, this study shows that AR suppression enhances CCL20 secretion and that the CCL20‐CCR6 axis promotes the migration of prostate cancer cells. Thus, the CCL20‐CCR6 axis, along with the AKT pathway, could be a new therapeutic target for prostate cancer.

https://onlinelibrary.wiley.com/doi/10.1111/cas.15683

